# First Occurrence of Fatal Intoxication Involving Methiodone (IC‐26) in Finland; a Medicolegal Autopsy Case Report

**DOI:** 10.1002/dta.70148

**Published:** 2026-09-11

**Authors:** Lasse Pakanen, Raimo A. Ketola, Pirkko Kriikku

**Affiliations:** ^1^ Forensic Medicine Unit Finnish Institute for Health and Welfare Oulu Finland; ^2^ Research Unit of Biomedicine and Internal Medicine, Medical Research Center (MRC) Oulu University of Oulu Oulu Finland; ^3^ Forensic Chemistry Unit Finnish Institute for Health and Welfare Helsinki Finland; ^4^ Department of Forensic Medicine University of Helsinki Helsinki Finland

**Keywords:** fatal intoxication, medicolegal autopsy, methiodone, novel synthetic opioids, opioid agonist treatment

## Abstract

Methiodone (IC‐26) is a non‐fentanyl novel synthetic opioid which has recently been detected in drug seizures in Europe. There is still limited data about the drug in published toxicological case work, both in non‐fatal and fatal cases. We present a medicolegal autopsy case report on a fatal intoxication in an individual undergoing opioid agonist treatment. A methiodone concentration of 1.5 mg/L was detected in the femoral vein blood and was considered the primary intoxicant causing death. This is the first published occurrence of fatal intoxication involving methiodone in Finland and emphasizes the need for constant monitoring of substance abuse during substitution therapy, including the potential use of novel synthetic opioids.

## 1 Introduction

Novel synthetic opioids constitute a rapidly growing group of substances found on illegal drug markets and causing deaths among drug users [1, 2]. Methiodone (IC‐26) is a non‐fentanyl novel synthetic opioid, an analog of methadone (Figure 1), a drug widely used in opioid agonist treatment. Although recognized as a substance of abuse [3], methiodone has only recently been identified in drug seizures in Europe [4].

**FIGURE 1:**
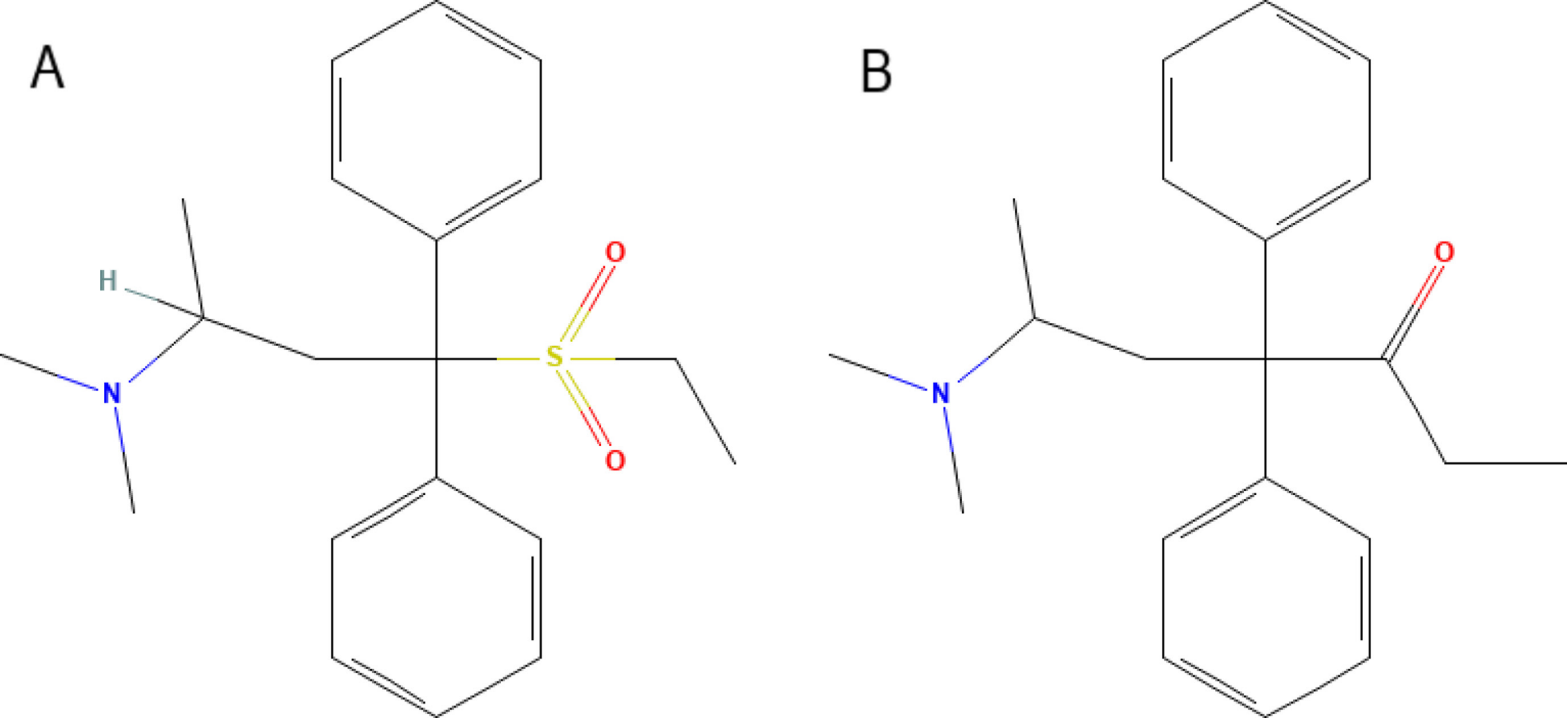
The chemical structure of methiodone (A) differs from that of methadone (B) with the sulfone group replacing the carbonyl group. Original figures acquired from PubChem database (methiodone, PubChem Identifier: CID 113832, https://pubchem.ncbi.nlm.nih.gov/compound/113832#section=2D‐Structure; methadone, PubChem Identifier: CID 4095, https://pubchem.ncbi.nlm.nih.gov/compound/Methadone#section=2D‐Structure).

To date, very little published data on methiodone is available. Four cases of non‐fatal methiodone intoxication have recently been reported in the Netherlands [5], and a further two cases in France [6]. German police reported two deaths related to the use of a “research chemical” methiodone in 2025 [7]. To the best of our knowledge, no further data on methiodone in toxicological case work is available in the scientific literature, and no detailed reports exist on fatal or non‐fatal intoxications related to the drug.

We present a medicolegal autopsy case involving fatal intoxication in which methiodone was considered the primary intoxicant. This is the first reported case of methiodone detection in Finland. A permit to review and report the medicolegal autopsy data was obtained from the Finnish Institute for Health and Welfare (document record number: THL/5621/6.02.00/2025).

## 2 Case History

A 30‐year‐old man was found dead in the bathroom of his apartment by a family member. The police discovered an envelope in the living room with references to Poland, along with small transparent plastic bags containing a white powdery substance. The bags were labeled “Tadalafil”, “Avizafone”, and “IC‐26 (Methiodone)”. “Not for human consumption” was also printed on the bags.

The deceased had a history of chronic narcotics use and chronic hepatitis C infection, and had been treated for anxiety symptoms and sleep problems. He was undergoing long‐term opioid agonist treatment, consisting of daily oral doses of methadone (dosage unknown) administered under controlled conditions at a health center. The deceased received his last dose of methadone two days before being found dead, and was not seen alive again after that visit. Previously, he had also complained of mild flu‐like symptoms.

The police ordered a medicolegal autopsy due to suspected accidental intoxication, in accordance with Finland's Act on the Investigation of the Cause of Death (459/1973) [8]. The complete autopsy revealed pulmonary edema as a nonspecific finding, but no apparent cause of death was determined. Samples were taken for toxicology, including femoral vein blood, urine, liver tissue, and vitreous humor.

The toxicological investigations were carried out at the Forensic Chemistry Unit of the Finnish Institute for Health and Welfare in Helsinki, Finland, in accordance with the routine protocol described earlier [9]. The analytical methods used are validated and enable the extensive detection and quantification of toxicologically relevant substances. In short: the urine sample was qualitatively screened using ultra‐high‐performance liquid chromatography coupled with time‐of‐flight tandem mass spectrometry (UHPLC‐TOF/MS/MS, Bruker Maxis Impact, Bruker Corporation). Concentrations in blood and urine were measured using UHPLC coupled with triple‐quadrupole mass spectrometry (UHPLC–MS/MS, Sciex Exion/5500 + QTRAP, A/B Sciex) (methiodone) and by gas chromatography–mass spectrometry (GC–MS, Agilent 6890/5675B or 6890/5973, Agilent Technologies) (methadone, 2‐ethylidene‐1,5‐dimethyl‐3,3‐diphenylpyrrolidine [EDDP], 7‐aminoclonazepam, and 11‐nor‐9‐carboxy‐tetrahydrocannabinol [THC‐COOH]). The toxicological findings are presented in Table 1.

**TABLE 1:** Results of toxicological investigation in the presented autopsy case.

Substance	Femoral vein blood concentration (mg/L)	Urine concentration (mg/L)
Methiodone	1.5	0.33
Methadone	0.42	1.7
EDDP	0.11	1.7
7‐aminoclonazepam	0.014	positive
THC‐COOH	0.0064	0.023
THC	negative	negative

Abbreviations: EDDP, 2‐ethylidene‐1,5‐dimethyl‐3,3‐diphenylpyrrolidine; THC, tetrahydrocannabinol; THC‐COOH, 11‐nor‐9‐carboxy‐tetrahydrocannabinol.

Following the completion of all the necessary ancillary investigations, the forensic pathologist who conducted the autopsy determined that the cause of death was fatal intoxication caused by methiodone, methadone, and clonazepam, with methiodone being the primary intoxicant. The manner of death was determined to be accidental.

## 3 Discussion

Although it is regarded as a novel synthetic opioid, methiodone was described in the literature as early as the late 1940s [10]. It was originally studied as a potential antitussive medication [11]. Only quite recently has methiodone become more widely available to people who use drugs through online vendors, where it is marketed mainly as a chemical for research purposes. Nonetheless, methiodone is widely considered an illegal substance [12]. It is also listed in the regulations on psychoactive substances prohibited in the consumer market under Finnish legislation [13]. Several discussions about methiodone as an intoxicant can be found on online forums for narcotics users, indicating increasing usage among these groups.

The effects of methiodone were initially thought to be comparable to those of methadone [10, 14]. Its addictive potential was suggested to be similar to that of morphine at single oral doses of 25–70 mg [11], which undoubtedly makes it a potent opioid. However, there is a lack of exact clinical data in humans, and no data on lethal dosages is available either. In the non‐fatal cases described previously, the two from France exhibited blood concentrations of 0.0187–0.0594 mg/L [6], whereas the four cases from the Netherlands had plasma concentrations ranging from 0.20–0.64 mg/L. [5] Although not directly comparable, the fatal case presented here had a notably higher concentration of 1.5 mg/L in the femoral vein blood. This was regarded as a lethal concentration, especially as it was paired with a methadone concentration of 0.42 mg/L, which corresponds to the median postmortem blood concentrations described earlier of 0.630 mg/L and 0.399 mg/L in cases of methadone‐only intoxications and multidrug intoxications, respectively [15]. According to the Forensic Chemistry Unit's database, the concentrations of methadone, 7‐aminoclonazepam, and THC‐COOH can be regarded as fairly moderate, as they all are equal to or lower than the mean and median concentrations of these substances detected in postmortem cases [16]. The same applies to EDDP, for which the recorded mean and median concentrations are 0.295 mg/L and 0.110 mg/L, respectively (unpublished data).

Methadone is considered to exhibit moderate PM redistribution at most [9, 17], but recent evidence suggests that atypical redistribution patterns may arise in chronic users [17]. Although no data exists as to the PM redistribution of methiodone, this should be taken into account given the similarity in the chemical structures of these compounds.

Opioid agonist treatment is associated with lower overall mortality, although the beginning of therapy is considered a high‐risk period [18]. Nonetheless, individuals undergoing opioid agonist treatment with methadone are at high risk of death from intoxication, especially when combined with the use of non‐prescription drugs [19, 20]. This risk was evident in the present case report, in which substitution therapy had been ongoing for several years. Hence, our report emphasizes the need for constant monitoring of substance abuse during therapy, including the potential use of novel synthetic opioids.

## Conflicts of Interest

The authors declare that they have no conflicts of interest.

## Data Availability

The data that support the findings of this study are available from Finnish Institute for Health and Welfare. Restrictions apply to the availability of these data, which were used under license for this study. Data are available from the author(s) with the permission of Finnish Institute for Health and Welfare.

## References

[1] M. P. Prekupec , P. A. Mansky , and M. H. Baumann , “Misuse of Novel Synthetic Opioids: A Deadly New Trend,” Journal of Addiction Medicine 11, no. 4 (2017): 256–265, 10.1097/ADM.0000000000000324. PMC5537029 28590391

[2] J. Yu , K. Diekhans , A. Tsang , and L. N. Rodda , “Fluorofentanyl and Novel Synthetic Opioids in Accidental Overdose Deaths,” Journal of Analytical Toxicology 48, no. 8 (2024): 573–581, 10.1093/jat/bkae062. 39087259

[3] B. Lovrecic , M. Lovrecic , B. Gabrovec , et al., “Non‐medical Use of Novel Synthetic Opioids: A New Challenge to Public Health,” International Journal of Environmental Research and Public Health 16, no. 2 (2019): 177, 10.3390/ijerph16020177. PMC6352208 30634521

[4] P. Dugues , T. Robin , M. Bellouard , et al., “Multi‐Analytical Identification of the Synthetic Opioids Cychlorphine and Methiodone (IC‐26) in Drug Seizures: First Detection in Europe,” Clinical Chemistry and Laboratory Medicine 64, no. 6 (2026): 1413–1424, 10.1515/cclm-2025-1428. 41507112

[5] E. J. Smolders , T. Klein , B. van de Velde , et al., “Methiodone (IC‐26): an emerging designer opioid causing severe toxicity,” [conference presentation]. NAFT Annual Meeting, Stockholm, Sweden, (May 6‐8, 2026).

[6] E. Le Carpentier , E. Lefebvre , E. Jaulin , et al., “À propos de deux cas de poly‐intoxication impliquant l'IC‐26,” Toxicologie Analytique et Clinique 38, no. 1 Suppl (2026): S35, 10.1016/j.toxac.2026.03.047.

[7] Die Bayerische Polizei , “Erneute Todesfälle und Intoxikationen in Zusammenhang mit ”Forschungschemikalien”,” accessed May 15, 2026, https://www.polizei.bayern.de/aktuelles/pressemitteilungen/093264/index.html.

[8] “Act on the Investigation of the Cause of Death [Laki kuolemansyyn selvittämisestä],” 459/1973, accessed August 17, 2026, https://www.finlex.fi/en/legislation/collection/1973/459.

[9] T. Launiainen and I. Ojanperä , “Drug Concentrations in Post‐Mortem Femoral Blood Compared With Therapeutic Concentrations in Plasma,” Drug Testing and Analysis 6, no. 4 (2014): 308–316, 10.1002/dta.1507. PMC4237191 23881890

[10] M. M. Klenk , C. M. Suter , and S. Archer , “The Preparation and Properties of Some Benzohydryl Sulfones,” Journal of the American Chemical Society 70, no. 11 (1948): 3846–3850, 10.1021/ja01191a090. 18102964

[11] A. B. Wolbach, Jr. and H. F. Fraser , “Addiction Liability of I‐C‐26,” Bulletin on Narcotics 1 (1963): 25–28, accessed May 15, 2026, https://www.unodc.org/unodc/en/data‐and‐analysis/bulletin/bulletin_1963‐01‐01_1_page005.html#bf004.

[12] M. Pardal , E. Wadsworth , and B. Kilmer , Illegal Synthetic Opioids: Can Europe Prevent a Crisis? (RAND Corporation, 2024), https://www.rand.org/pubs/perspectives/PEA3270‐1.html.

[13] “Government Decree on Psychoactive Substances Banned from Consumer Markets [Valtioneuvoston asetus kuluttajamarkkinoilta kielletyistä psykoaktiivisista aineista],” 1130/2014, accessed May 15, 2026, https://www.finlex.fi/en/legislation/2014/1130.

[14] D. Lednicer , “Medicinal Chemistry of Central Analgetics,” in Central Analgesics, ed. D. Lednicer (John Wiley & Sons, Inc., 1982), 137–213.

[15] G. Pelletti , V. Giunchi , P. Fais , et al., “Characterizing Post‐Mortem Blood Concentrations in Methadone‐Related Deaths,” International Journal of Legal Medicine 140, no. 1 (2026): 207–216, 10.1007/s00414-025-03620-0. PMC12808249 41060397

[16] R. A. Ketola and I. Ojanperä , “Summary Statistics for Drug Concentrations in Post‐Mortem Femoral Blood Representing All Causes of Death,” Drug Testing and Analysis 11, no. 9 (2019): 1326–1337, 10.1002/dta.2655. 31128576

[17] B. Garneau , C. Roy , J. Motard , B. Desharnais , C. Bouchard , and P. Mireault , “Atypical Postmortem Redistribution in Chronic Methadone Consumers,” Journal of Analytical Toxicology 48, no. 5 (2024): 343–349, 10.1093/jat/bkae016. 38491986

[18] L. Sordo , G. Barrio , M. J. Bravo , et al., “Mortality Risk During and After Opioid Substitution Treatment: Systematic Review and meta‐Analysis of Cohort Studies,” BMJ 357 (2017): j1550, 10.1136/bmj.j1550. PMC5421454 28446428

[19] C. U. Andersen , S. K. Hermansen , K. B. Olsen , K. Linnet , and K. W. Simonsen , “Fatal Poisoning Among People Who Use Drugs in Denmark in 2022,” Danish Medical Journal 72, no. 11 (2022): A03250147, 10.61409/A03250147. 41133335

[20] N. C. Stam , Y. Bonomo , J. Furler , O. H. Drummer , and J. L. Schumann , “Drug Toxicity Deaths Associated With Opioid Agonist Treatment: An Analysis of Methadone and Buprenorphine‐Related Fatalities in Victoria, Australia,” Forensic Science International 379 (2026): 112772, 10.1016/j.forsciint.2025.112772. 41406752

